# Enhanced Schemes for Brine Valorization via Electrodialysis with Bipolar Membranes Powered by Renewable Energy

**DOI:** 10.1021/acsomega.4c08609

**Published:** 2025-03-07

**Authors:** Calogero Cassaro, Giovanni Virruso, Andrea Cipollina, Adriano Fagiolini, Alessandro Tamburini, Giorgio Micale

**Affiliations:** Dipartimento di Ingegneria, Università degli Studi di Palermo, Viale delle Scienze Edificio 6, Palermo 90128, Italy

## Abstract

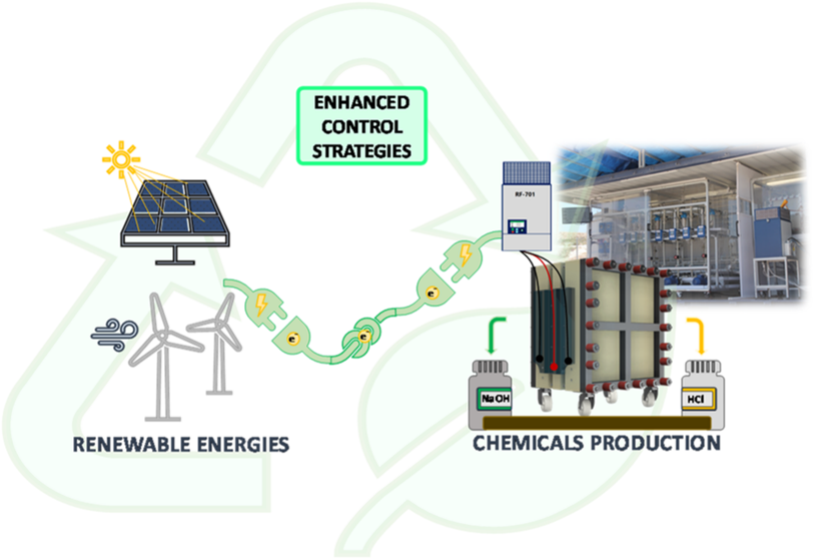

Powering water treatment technologies with renewable energies by using the process buffering capacity as a way to indirectly store energy has been recently proposed as an effective strategy for the smart use of energy. With this respect, the production of chemicals from waste brines via electrodialysis with bipolar membranes (EDBM) can be particularly suitable due to its high energy intensity along with the extreme flexibility of the process. This study demonstrates, through real-environment experiments at the pilot scale, the feasibility of coupling an EDBM pilot plant with renewable energies (solar). The pilot plant was tested in a continuous process configuration (feed and bleed mode) under two different irradiation scenarios, i.e., summer and winter. The use of the controllers implemented allowed us to maintain the target concentration for acid and base fixed at 0.5 mol L^–1^ in both scenarios. In the summer scenario, current efficiency (CE) values higher than 90% and specific energy consumption (SEC) values lower than 2 kWh kg^–1^ were obtained, still maintaining a specific productivity (SP) of about 0.2 kg h^–1^ m^–2^. In the winter scenario, a current efficiency >80% was obtained, while SEC and SP values up to 1.6 kWh kg^–1^ and 0.06 kg h^–1^ m^–2^ were found, respectively. Results suggest that EDBM technology is perfectly suitable for the valorization of waste brines by using green energy sources, thus paving the way for its development at an industrial scale.

## 1 Introduction

Exploitation of renewable energy sources (RES)^1^ and mitigation of water scarcity^2^ represent two of the main challenges of the 21st Century. European Commission has proposed scaling up renewable energy utilization in power generation, industry, buildings, and transport up to 45% by 2030.^3^ According to this scenario, coupling of RES and the traditional electric grid is considered a reliable alternative to maintaining grid stability while satisfying energy demand.^4^ However, RES such as solar and wind are weather-dependent and exhibit the weakness of not being capable of following the grid request variation. The implementation of energy storage systems (ESS) can overcome this limitation playing a significant role in power generation by supporting the different energy sources^5^ to meet the requirements of loads.^6^ The development of smart grid^7^ would allow to incorporation of information and communication technologies into every aspect of electricity generation, delivery, and consumption, thus playing a significant role in power generation by balancing the different energy generation and utilization devices to meet the requirements of the grid.^8^ The energy storage systems (ESS) can be classified into several categories, according to the form of the energy stored, such as thermal, mechanical, chemical, electrochemical, and hybrid,^9^ converting energy from one form (mainly electrical energy) to a storable form^10^ and reserving it in various mediums which can be converted back into electrical energy when needed.^11^ Among the ESS, the chemical energy storage systems involve all technologies where the electrical energy is used to store the energy as chemical compounds.^12^ Recently, several works in the literature have proposed different approaches to store and valorize energy via freshwater production.^13,14^ Porrazzo et al.^15^ studied a membrane distillation (MD) system powered by solar energy, as a thermal source, under real meteorological conditions. They developed an optimized feedforward control system based on a neural network model of the plant. Through the use of the control system, they managed to maximize the production of distillate by the available energy. Campione et al.^16^ studied the behavior of an electrodialysis (ED) stack supplied by renewable energy sources, such as solar and wind. They designed a hybrid, feedforward-feedback control system capable of keeping the target product concentration constant, independent of the available power variations. A PV-ED experimental setup was tested at Tanote, in the Thar desert region by Adiga et al.^17^ They successfully utilized a PV panel of 0.45 kW to power an electrodialysis stack at the pilot scale, able to produce 1 m^3^ of freshwater per day. The coupling of the electrodialysis system with solar panels was further tested by Cìrez et al.^18^ demonstrating the flexibility of a batch ED supplied by different PV configurations. The integration of RO-ED units with renewable energy systems has been thoroughly analyzed by Nurjanah et al.^19^ to address the challenges associated with the high energy demands of the reverse osmosis process. Their findings suggest that control systems can improve process performance by regulating the high-pressure pump and dosing the use of battery storage in response to fluctuations in renewable energy source. In recent years, electrodialysis with bipolar membranes (EDBM or BMED) has been proposed^20^ as a reliable technology to store energy in the form of chemicals such as acids and bases.^21^ The EDBM technology is, in principle, suitable to be powered with highly time-varying energy such as that provided by renewable energy sources.^22^

Electrodialysis with bipolar membranes (EDBM) is an electro-membrane process,^23^ which allows the conversion of electric energy into the production of chemicals with high added value (acids and bases), starting from the corresponding salts.^24^ An EDBM stack is characterized by the repetition of many units called triplets.^25^ A triplet consists of a sequence of three ion-exchange membranes (IEMs),^26^ namely, cationic, bipolar,^27^ and anionic, and three channels: basic, acid, and salt channel (Figure S1). The key element of an EDBM unit is the bipolar exchange membrane (BPM).^28^ This membrane is a novel ion-exchange membrane (IEM) constituting a layer of anion exchange material (AEL) and a layer of cation exchange material (CEL) welded together.^29^ The zone between CEL and AEL is named “interlayer” and is often supplemented with catalysts to promote water dissociation.^30^ Through the application of an external electric field, cations and anions dissolved in the saline solution fed to the unit migrate selectively toward the cathode and the anode, respectively, encountering the protons and hydroxyl ions generated inside the bipolar membrane in the acid and base channel, respectively.^31^

The production of acids and bases via EDBM has been widely proposed in the literature as an attractive option,^32^ compared to conventional industrial processes, for its high environmental sustainability^33^ (since it valorizes waste brines). Moreover, the utilization of RES could allow to overcome the energetic issues exploiting the surplus grid-load through the storage of this energy as chemicals with high added value.^34^ Herrero-Gonzalez et al.^35^ implemented a PV-EDBM setup, coupling a lab-scale EDBM stack (*A*_m_ = 0.01 m^2^) with a PV solar array simulator. A SCADA system was used to control the plant. The system was operated in a semibatch configuration managing the outlet flow rates through an on–off control of the pH of the acid solution and an on–off control for the conductivity of the saline solution. They demonstrated that the use of the control system can lead to a reduction of the specific energy consumption (SEC) of the acid from 7.3 kWh kg_HCl_^–1^ (at fixed current density) to 4.4 kWh kg_HCl_^–1^ (at variable current density) adapting the flow rate of the feed and of the acid stream according to energy availability. The techno-economic feasibility of integrating EDBM with solar panels was further analyzed by Herrero-Gonzalez et al.^36^ They found that the levelized cost of sodium hydroxide was lower when using PV panels since it eliminated the electricity cost of the grid mix. Moreover, they stated that the PV-EDBM integrated process significantly reduced the emissions and, consequently, the costs associated with the carbon tax and EU Emissions Trading System allowances. Only a few studies have been conducted in this area, and these have generally focused on feasibility rather than on technology assessment within real demonstration plants.^37^ Additionally, while enhanced process control strategies and their potential impact on EDBM performance and operations have been suggested,^38^ they still have to be fully explored. The relevance of these strategies to the “smart” utilization of nonstationary power from renewable energy sources also remains to be investigated.

This work aims to demonstrate the key role of EDBM technology in sustainable perspectives where the highly fluctuant surplus energy, coming from renewables, can be efficiently stored and converted in the form of highly valuable chemical reagents (i.e., acid and alkaline products). To reach this purpose, enhanced single-loop control strategies were developed and tested first through the dynamic simulation toolbox of MATLAB (Simulink). Subsequently, the controllers were implemented in the LabVIEW environment and tested in the real plant^39^ with a PV solar simulator. The goal of the control system is to allow continuous operation of the equipment in highly transitory regimes, aiming to reach and maintain the set-point concentration for both acid and base. At the same time, the controller ensures stability over time, maximizing the use of the available energy in terms of performance parameters.

## 2 The EDBM Pilot Plant and Its Enhanced Control Strategies

### 2.1 The Feed and Bleed Configuration

**Figure 1:**
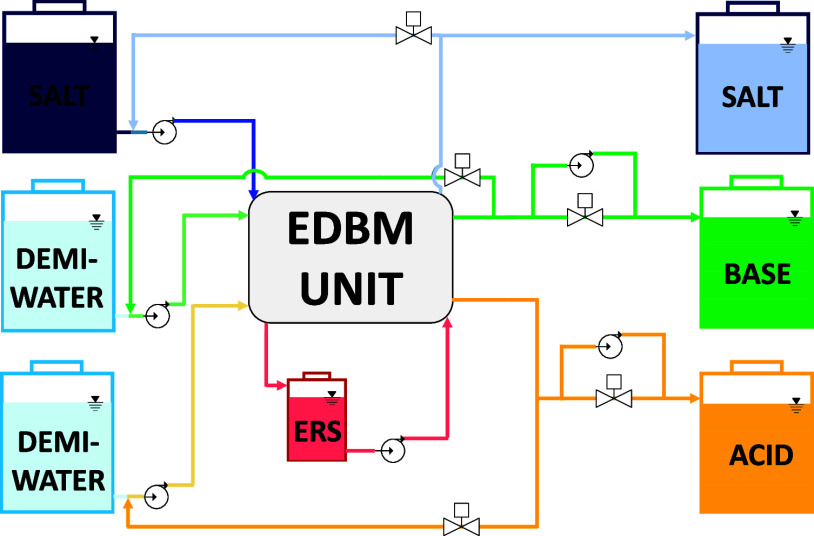
Process flow diagram of the EDBM pilot operated in feed and bleed mode (continuous operation mode).

The EDBM pilot plant was equipped with all of the instrumentations needed for control purposes. Moreover, the unit was operated by feeding continuous acid, base, and salt solutions, while the electrode rinse solution was operated in closed-loop mode.^40^ A simplified process flow diagram (PFD) of the feed and bleed configurations is reported in Figure 1.

The purpose of the employed layout was to make the plant as flexible as possible in terms of controllability, guaranteeing continuous production and long-term stability. The choice of operating all lines in feed and bleed guaranteed the possibility of setting the molar ratio between salt and base (which represented the product of interest), allowing it to vary for the process needs. With this respect, a key role was played by electro-actuated valves and gear pumps both installed in the outlet lines, which allowed us to fine-tune the outlet flow rate.

### 2.2 EDBM Equipment and Membranes

The EDBM stack was an FT-ED1600-3 unit provided by FuMA-Tech GmbH (Germany). The stack consists of 40 triplets divided into two modules with 20 triplets each, reaching a total active membrane area of 19.2 m^2^. The stack is assembled with two opposite cathode plates and a shared anode, with an internal fluid distribution generating a 2-stage operation. The unit is provided with the following ionic exchange membranes: FUMASEP FAB-PK anion exchange membranes, FUMASEP FKB cation exchange membranes, and FUMASEP FBM bipolar membranes (Germany). A comprehensive list of properties of the ion-exchange membranes composing the stack is reported in the Supporting Information (Table S1). Each membrane has an area of 0.454 × 0.345 m^2^. Woven spacers 350 μm thick and made of polypropylene were used to separate the IEMs and give dimensional stability to the channels hosting the solutions. The anode and cathode are DSA and stainless steel, respectively.

### 2.3 Sensors and Control Devices

Magnetic induction flowmeters were used to measure stack inlet and outlet flow rates. The conductivity and pH values of solutions were monitored using inductively conductive sensors and pH sensors, respectively. Pressure transducers were also installed in the inlet and outlet lines of the unit to evaluate the pressure drops across the stack. Magnetic-driven centrifugal pumps equipped with an external inverter, gear pumps with integrated inverters and electro-actuated valves were adopted as final control element to manage the process control variables (more details about the sensors and the final control elements employed in the pilot plant are reported in the Supporting Information, Table S2, where Figure S2a shows a schematic representation of the pilot system, along with sensor and control devices while a screenshot of the Human Machine Interface (HMI) is reported in Figure S2b).

### 2.4 Operating Procedures under Dynamic Conditions

Before the control systems were designed, the analysis of the transient behavior of the uncontrolled system was carried out to determine the dynamic features of the plant. To this aim, an extensive experimental campaign was conducted to cover the entire operating range of the EDBM stack, which, having a nonlinear behavior, exhibited different responses depending on the operating conditions adopted. All of the tests were performed by feeding 1 mol L^–1^ of Sodium Chloride solution (NaCl >99.5% purity, Saline di Volterra S.r.l with impurities of Ca^2+^ and Mg^2+^ <10 ppm) in the salt compartment while using RO-permeate (conductivity <350 μS cm^–1^) as a feed for the production of acidic and alkaline solutions. On the other hand, a solution of 0.125 m^3^ with a concentration of 0.25 M of Na_2_SO_4_ (technical grade, CR GRUPO CRIMIDESA) was used for the ERS. For the analytical characterization of the products, acid, and base samples of 50 mL were collected once per hour, using titration of Na_2_CO_3_ (0.05 M) and HCl (0.1 M), respectively, employing methyl orange as an indicator. Table 1 shows a summary of the main dynamic tests performed.

**Table 1:** Summary of the Main Dynamic Tests Performed in the Pilot Scale EDBM Unit

test no.	inlet variable	step size	outlet variable	purpose
1	the voltage of the inlet pump	±25%	inlet flow rate	design of the inlet flow rate control
		±50%		
2	the voltage of the inlet pump	±25%	inlet pressure	design of the inlet pressure control
		±50%		
3	degree of the opening of the electro-actuated valve	±25%	outlet flow rate	design of the outlet flow rate control
		±50%		
4	the voltage of the outlet pump	±25%	outlet flow rate	design of the outlet flow rate control
		±50%		
5	outlet flow rate	±25%	outlet conductivity	design of composition control
		±50%		
6	current density	±25%	outlet conductivity	study of the disturbance on outlet conductivity
		±50%		
7	applied voltage	±25%	power	design of the DC drive control
		±50%		

**Figure 2:**
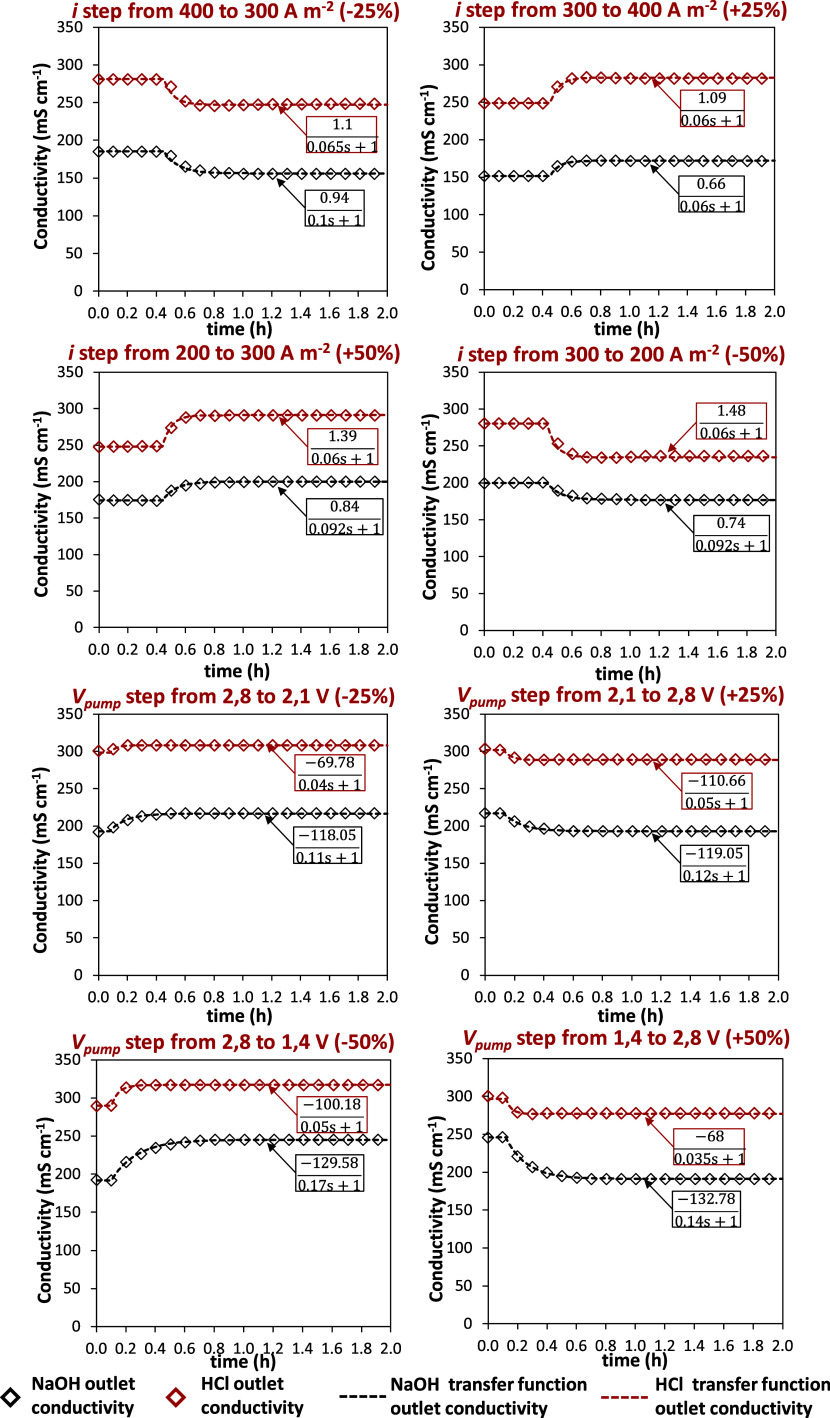
Dynamic response of the uncontrolled system to a step variation of current density (*i*) and outlet flow rate (*V*_pump_). Symbols: experimental data; solid line: Simulink Transfer Function trend.

Figure 2 shows the dynamic response of the acid and base conductivities as a consequence of different step variations in current density and output flow rate, respectively. Several tests were performed starting from steady-state conditions of 200 and 400 A m^–2^. In particular, from Figure 2, it was possible to observe how a disturbance of the same size led to different steady-state values and time constants for the outlet conductivities. Moreover, a faster response of the acid was observed in most cases. To obtain a reliable relationship between the inlet and the outlet variables, all of the other variables of the process were kept constant and equal to the value of steady-state value. All of the dynamic trends were fitted using first-order or first-order plus dead time transfer functions. From these transfer functions, it was possible to create a linearized model of the process in a wide range of operating conditions thus allowing the design of all plant controllers.

### 2.5 Enhanced Single-Loop Control Schemes Design and Tuning

#### 2.5.1 Design of the Recirculation Flow Rate-Maximum Pressure Using Override Logic

**Figure 3:**
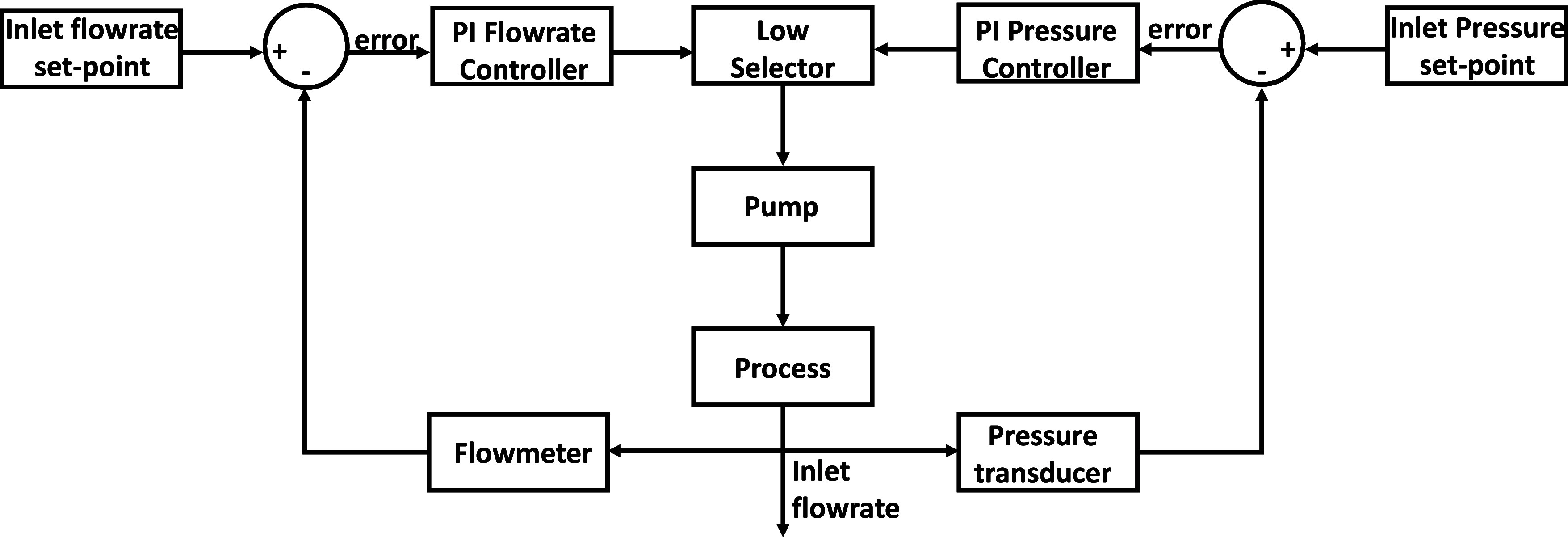
Control scheme between the recirculation flow rate-maximum pressure with override logic.

The dynamic trends collected from the experimental campaign were fitted using the transfer function model to obtain a reliable relationship having the outlet variables as a function of the inlet ones. The links between the pump command signal and the recirculated flow rate as well as the relationship between the pump control signal and the recirculated pressure were described using a first-order transfer function. In particular, in test no. 1 (Table 1), values of 2.3 L min^–1^/V and 6 × 10^–4^ h (2.2 s) were found for the gain and the time constant, respectively. Conversely, values of 0.35 barg/V and 4.5 × 10^–4^ h (1.6 s) were obtained in test no. 2. The transfer functions obtained from both tests were used to design an enhanced control scheme, enabling control of the recirculated flow rate and inlet pressure through an override logic. In Figure 3 the control scheme is reported showing the feedback control loops for both controlled variables, flow rate and pressure. In this scheme, the flow rate and the pressure of the recirculated stream were read and sent to the comparators of the flow rate and pressure feedback loop to obtain the error concerning the desired values. The errors were processed from the two controllers and sent to the low signal selector, which chose the lower value among these. The lower controller signal, subsequently, varied the rpm of the inlet pump (manipulated variable), leading to the corresponding values of flow rate and pressure. For both controllers, a proportional-integral (PI) action was selected to delete the offset. The internal model control (IMC) was selected as a tuning method to obtain preliminary values of the proportional and integral action. These values were then refined through a fine-tuning approach to have a robust response. After the fine-tuning, the proportional actions resulted equal to 0.10 and 3.05 for the flow rate and pressure controller, respectively, while the integral constant time was selected equal to 8 × 10^–4^ h (2.9 s) in both cases. This control strategy was implemented for safety reasons to prevent the EDBM stack from overpressure during the long-term operations. The override scheme was designed to employ the lower control signal from the flow rate controller enabling the pressure controller in case the maximum pressure was reached. As a consequence, the pressure control took over fixing the inlet pressure to the maximum allowed value until the nominal condition was restored.

#### 2.5.2 Design of the Product Quality Control

**Figure 4:**
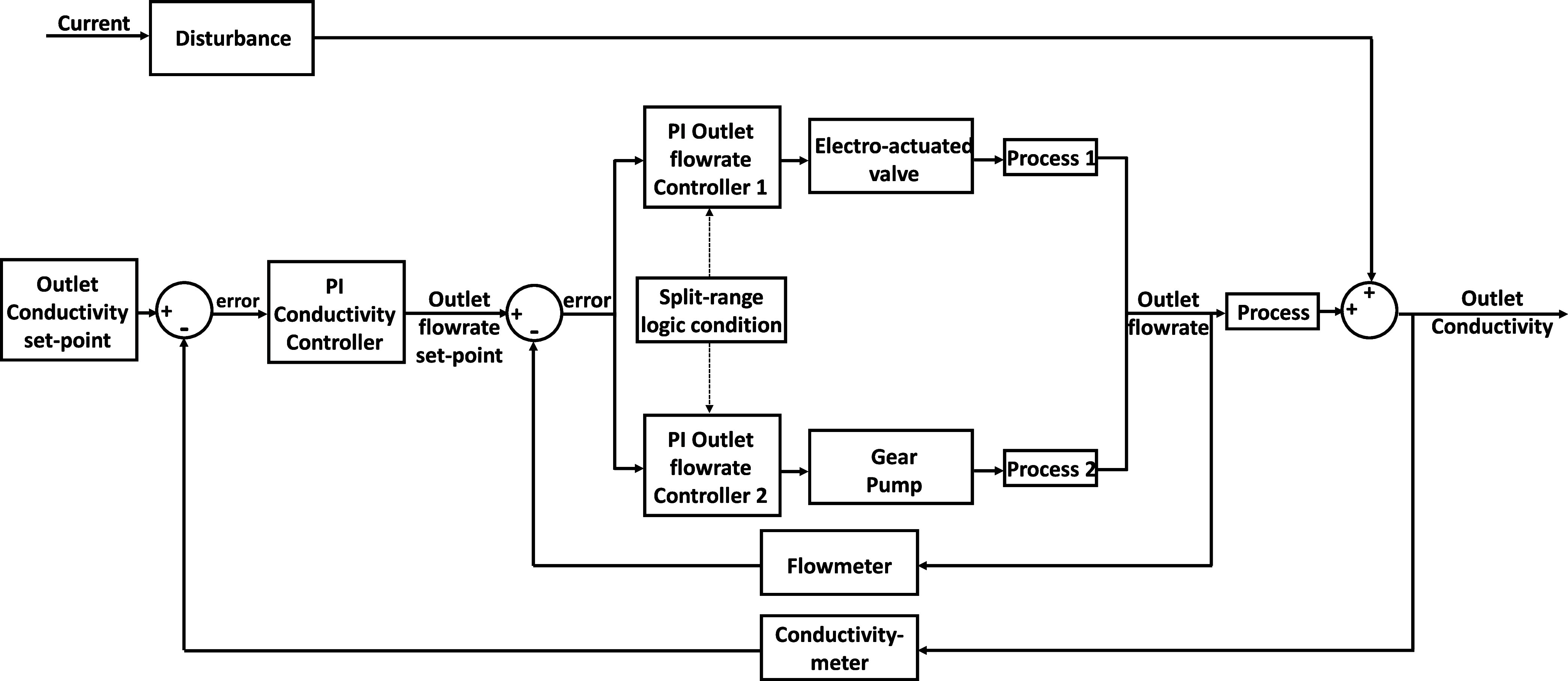
Scheme of the cascade controller of the outlet conductivities with split-range logic for the outlet controller.

The design and implementation of the quality control loop are key points of this work. The dynamic test nos. 3, 4, 5, and 6 reported in Table 1 were analyzed and fitted using the transfer function model to derive a model of the process being reliable in a wide range of operating conditions. The first-order transfer functions model was employed to describe the dynamic responses of the outlet conductivities with flow rate and current density since all trends exhibited qualitatively the same shape. These data were utilized to design a cascade control, adopting two feedback control loops. The master controller was a conductivity control loop, while the secondary one was an output flow rate controller with split-range logic. The transfer functions linking the outlet flow rate with the voltage of the gear pumps (test no. 4) were characterized by a gain of 0.31 L min^–1^/V and a time constant equal to 5.5 × 10^–4^ h (2.0 s). Concerning the electro-actuated valve (test no. 3), values of 0.06 L min^–1^/% of opening and 9 × 10^–4^ h (3.2 s) were found for gain and time constant, respectively. Figure 4 shows the split-range logic of the slave controller, used to regulate the outlet product flow rate. The outlet flow rate was read through a magnetic flowmeter and was compared with the set-point value. The split-range logic condition received the flow rate set-point from the master controller enabling either the outlet flow rate controller 2, when the value was lower of the split-range condition set at 1.2 L min^–1^, or both controllers whenever the set-point value was higher than 1.2 L min^–1^. Proportional-integral controllers were chosen to regulate the outlet flow rate by using the internal model control as a tuning method. The controller parameters were adjusted through fine-tuning, leading to a proportional coefficient of 0.23 and 2.0 and to an integral time constant of 2 × 10^–3^ h (7.2 s) and 4 × 10^–4^ h (1.4 s) for the pump and the electrically actuated valve, respectively.

The transfer functions obtained from tests no. 5 and no. 6 were employed to design the master controller. The gain and the time constant showed the nonlinear behavior of the output variable considering the same step size of the input. In particular, for test no. 6 describing the conductivity of the product as a function of the current density, values in a range of 0.74–1.56 mS cm^–1^/A m^–2^ were obtained in terms of gain of the base stream with a time constant ranging from 6 × 10^–2^ h (3.6 min) to 0.14 h (8.4 min). Different values were found for the acid solution exhibiting a gain between 1.09 and 2.58 mS cm^–1^/A m^–2^ and a time constant in a range of 6 × 10^–2^ h (3.6 min) to 0.14 h (8.4 min). The nonlinear behavior of the system was also confirmed by the transfer functions linking the outlet flow rate which was chosen as manipulated variable, with the conductivity of acid and base. In this case, the gain resulted in a range of −53.35 ÷ −200 to −68 ÷ −123 mS cm^–1^/L min^–1^ for base and acid, respectively. In terms of time constant, similar values in a range of 6 × 10^–2^ h (3.6 min) to 0.18 h (10.8 min) were obtained. Figure 4 shows the implemented scheme for the master controller. The outlet conductivity is compared with the set-point value, and the resulting error is sent to the primary controller. Subsequently, the control action is sent to the slave controller fixing the set-point of the outlet flow rate. Due to the nonlinear behavior of the EDBM unit, for the master controller, 12 different PI controllers, for both base and acid, were designed using the internal model control method. The final parameters were calculated as an average of all controllers to make the control action as flexible as possible in the desired range of current densities between 50 and 500 A m^–2^. Finally, the proportional gain for the base master controller was equal to −6 × 10^–3^ with an integral time constant of 8.4 × 10^–2^ h (5.0 min). The parameters of the acid master controller were quite similar and equal to −9 × 10^–3^ and 9.6 × 10^–2^ h (5.76 min) for the proportional and integral actions, respectively.

#### 2.5.3 Design of the Ratio Control Scheme

**Figure 5:**
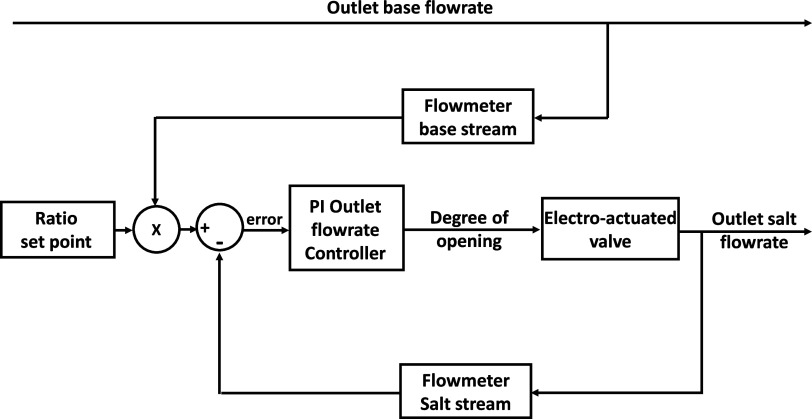
Ratio control strategy between salt and base outlet flow rates.

To improve the performance of the quality controller, another important step was the design of a ratio control scheme between the alkaline and salt streams. The effect of the outlet salt stream with the degree of opening of the feed and bleed valve was described with a first-order transfer function having a gain of −6 × 10^–2^ L min^–1^/% of opening and a time constant of 8.9 × 10^–4^ h (3.2 s). Figure 5 reports the scheme of the ratio control loop implemented in the Programmable Logic Controller (PLC). The outlet flow rate of the alkaline solution is read through a flowmeter and sent to a multiplier. Here, the base flow rate is multiplied by a factor equal to the ratio set-point, fixing the set-point flow rate of the salt feedback loop. To regulate the flow rate of the salt, a PI controller designed by using the Internal Model Control (IMC) method was chosen. After online fine-tuning, the final values resulted equal to −1.5 and 9 × 10^–4^ h (3.2 s) for the proportional gain and the integral action, respectively.

#### 2.5.4 Design of the DC Drive Control for PV Maximum Power Point Tracker (MPPT)

**Figure 6:**
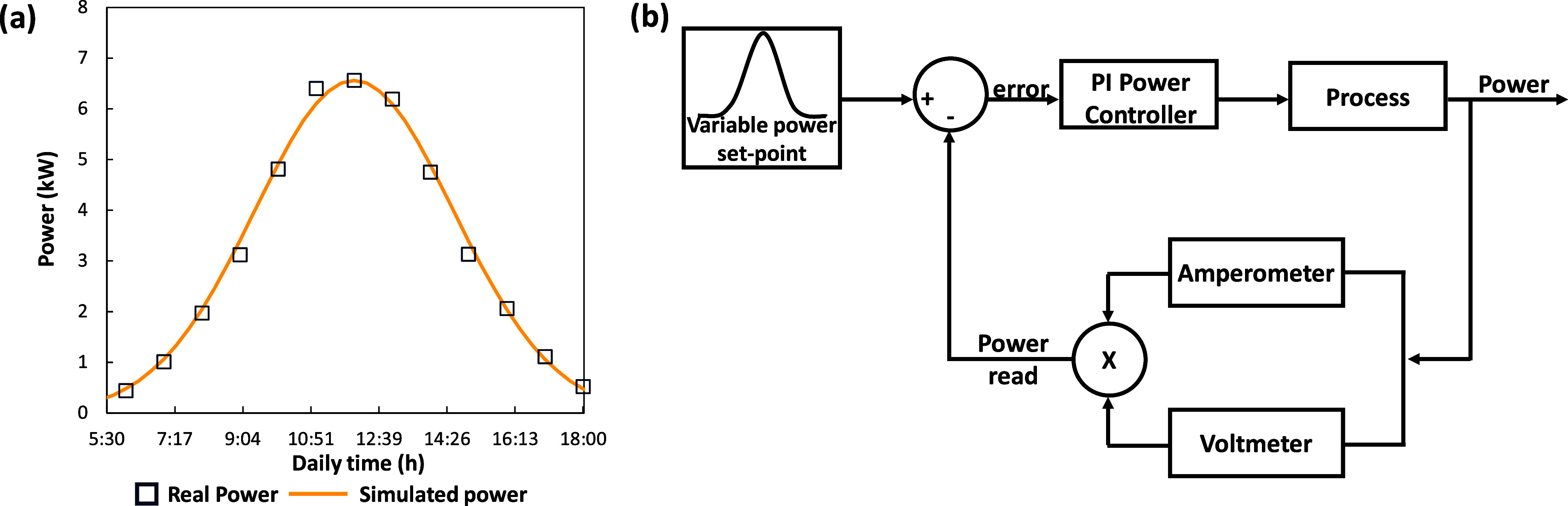
(a) Comparison between a real solar radiation curve on a summer day and the simulated power curve. (b) Feedback control scheme of the DC drive power supply to the EDBM stack.

To make the EDBM unit able to work under highly transitory regimes, the latter part of the design phase regarded the implementation of a DC drive control for photovoltaic maximum power point tracker (MPPT).^41^ This control scheme is thought to operate with a dynamic set-point through the use of a Gaussian function fitting the real power coming from a solar field. Two irradiation curves, representing the summer (July) and winter (December) seasons in Lampedusa Island (where the pilot is installed), were taken from the European PV-GIS platform.^42^ From the irradiation data, a small solar field was designed assuming to utilize a commercial panel with 21% efficiency (JA SOLAR, type JAM72S30-540/MR) and a surface area of the solar field equal to 32 m^2^ to have a peak power of 6.5 kWp. Figure 6a shows the trend of the simulated power (yellow line), in comparison with the real one (blue squares), utilized as a set-point for the control logic. With this respect, the power provided from the DC drive to the EDBM stack was chosen as the controlled variable, while the applied voltage was adopted as the manipulated variable. The current density was able to vary according to the resistance of the stack depending on spacers, membranes, and the concentration of the solutions inside the channels. The dynamic trend linking power and voltage was described assuming just a gain equal to 0.29 kW/V (test no. 7), without a time constant. In Figure 6b the feedback control loop implemented for the DC drive is depicted. The power provided to the stack was obtained by multiplying the current and the voltage read through the amperemeter and voltmeter, respectively. The power read was subsequently compared with the dynamic set-point, resulting in an error provided to the control low. A proportional-integral controller was selected to track the power and the final parameter, after fine-tuning, at 1 and 2.5 × 10^–3^ h (9 s) for the proportional and the integral actions, respectively.

### 2.6 Performance Indicators

Different performance indicators were employed to analyze the performance of the EDBM unit:

Current efficiency (CE, %) accounts for the amount of the electric charges supplied to the system that were successfully converted into the production of protons or hydroxide ions. Equation 1 shows the formula employed for the current efficiency calculation
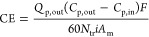
1where *C*_p,out_ and *C*_p,in_ refer to the outlet and inlet concentrations (mol L^–1^) of product, respectively; *Q*_p_ represents the outlet flow rate (L min^–1^) of sodium hydroxide or hydrochloric acid streams; *F* is Faraday’s constant (i.e., 96,485 C mol^–1^); *N*_tr_ is the triplet number; *A*_m_ is the active membrane area (m^2^); and *i* (A m^–2^) is the electric current density provided to the stack.

Specific energy consumption (SEC, kWh kg^–1^) is the energy consumed to produce 1 kg of the desired product (i.e., either NaOH or HCl). Equation 2 shows the formula utilized for the specific energy consumption calculation and its relationship with current efficiency
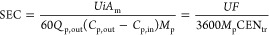
2where *U* is the electric potential (V) applied to the stack and *M*_p_ is the molar mass of the desired product (g mol^–1^), while the other variables meaning is the same as above.

Specific Productivity (SP, kg h^–1^ m^–2^) indicates the mass of product (i.e., either NaOH or HCl) produced in 1 h of operation per unit of total membrane area. Equation 3 shows how the specific productivity was determined and its relationship with current efficiency and current density
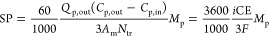
3where the variables’ meanings are the same as above.

## 3 Results and Discussion

### 3.1 Testing of the Enhanced Control Systems

#### 3.1.1 Testing of the Override and Ratio Control Logic

**Figure 7:**
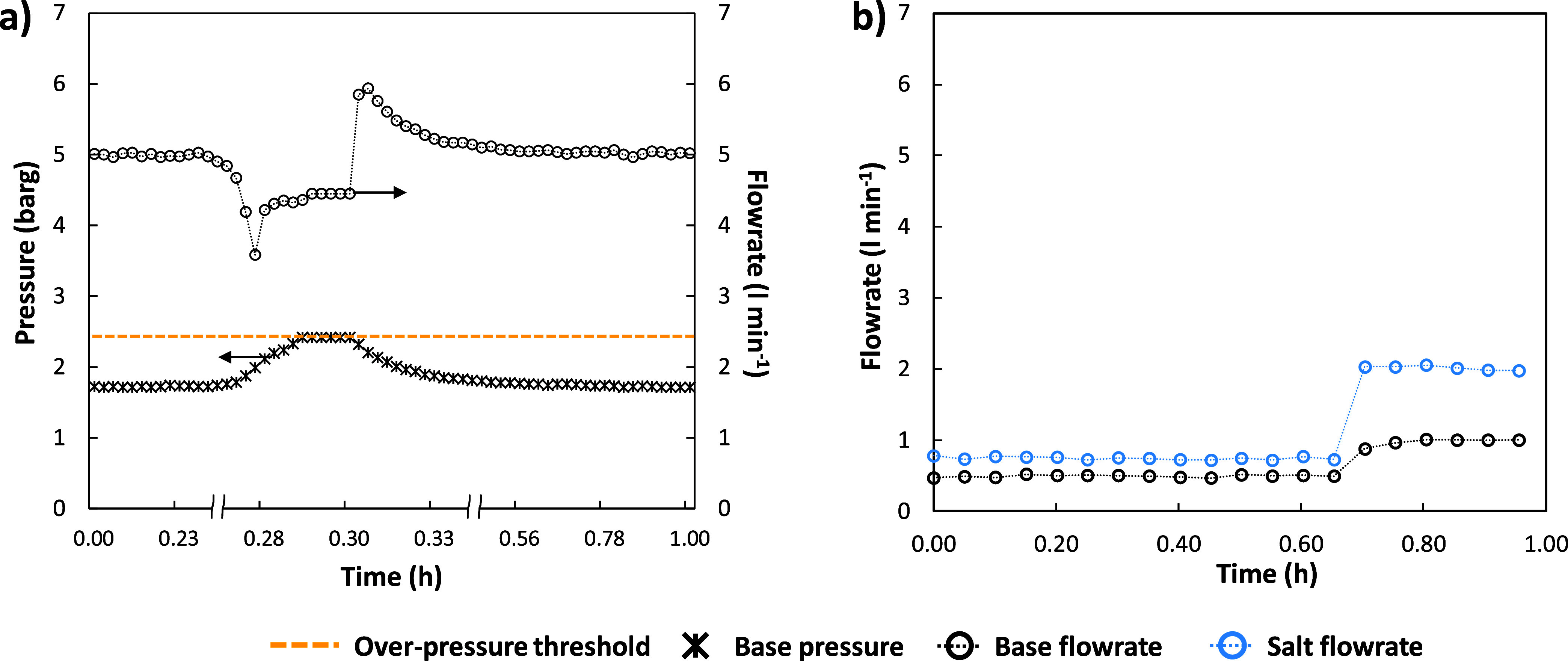
(a) Functioning example of the override controller (a zoomed-in *x*-axis is used in the zone where the pressure-limited condition is achieved). The dashed line represents the pressure threshold over which the system cannot operate. (b) Functioning example of the ratio controller, with a set-point ratio of 1.5 in the first 0.65 h and 2 in the remaining part of the test, within a variable flow rate regime.

After the design phase, the enhanced controllers were tested in a real plant. The override control was designed to prevent potentially dangerous clogs inside the channels. To verify his effectiveness, a manual valve was employed to mimic fouling and scaling phenomena, leading to the activation of the override logic. Figure 7a shows the trends of the inlet flow rate and pressure of the base stream during the test. In the first part of the test, the system worked in nominal conditions, with the recirculated flow rate fixed at 5 L min^–1^ using the flow rate controller and a pressure equal to 1.7 barg. Once the manual valve was partially closed, the flow rate decreased abruptly. Consequently, the flow rate controller tried to bring the controlled variable to the desired value leading to a corresponding increase of the inlet pressure. When the pressure reached the threshold value (dash yellow line), the flow rate controller was replaced by pressure one, keeping the pressure equal to the overpressure value set at 2.4 barg (the maximum allowed from the provider was 3 barg). Then, the valve was opened causing a quick increase in the inlet flow rate and a decrease in the inlet pressure below the override value. The flow rate control was reactivated thereby restoring the nominal operating condition for both flow rate and pressure.

Figure 7b shows testing of the ratio control implemented between the salt and the base outlet streams. Initially, the outlet alkaline flow rate was fixed at 0.5 L min^–1^ and the ratio was kept equal to 1.5 corresponding to an outlet salt flow rate of 0.75 L min^–1^. In the second part of the test, the flow rate of the base and the ratio with the saline stream were raised to 1 L min^–1^ and 2, respectively, leading to an increase in the output salt stream up to 2 L min^–1^.

#### 3.1.2 Testing of the Cascade Control with Split-Range Logic

**Figure 8:**
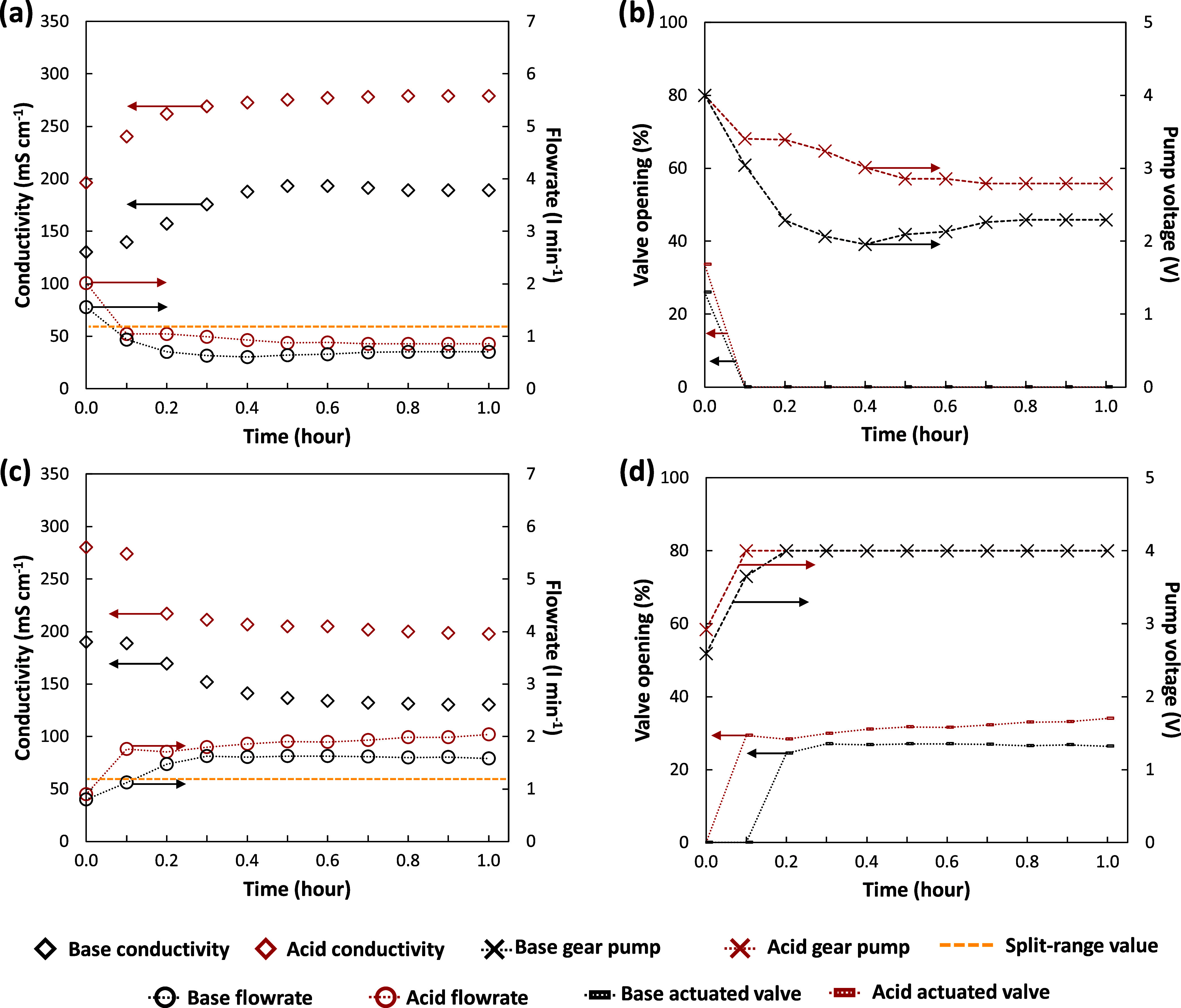
(a, b) Response to a +30% set-point variation of the outlet conductivities, the corresponding trend of the control variables (outlet flow rates), and relevant control signal variation to the electro-actuated valve and gear pump. (c, d) Response to a −30% set-point variation of the outlet conductivities, the corresponding trend of the control variables (outlet flow rates), and relevant variation of the control signal to the electro-actuated valve and gear pump. Dashed yellow lines indicate the flow rate value at which the control spit takes place.

To assess the performances of the quality controllers, different tests were performed for both set-point changes and disturbance response. Figure 8a represents a real response of the system to a set-point variation of +30% of acid and base outlet conductivities (controlled variables) with the corresponding trends of the outlet product flow rates (manipulated variables). A faster response of the acid conductivity was observed compared to that of the base. In particular, the response time resulted in 12 and 18 min for acid and base, respectively, due to the higher proportional gain of the acid controller. The outlet flow rate of the acid stream varied quickly and was near the steady-state conditions. On the other hand, the outlet flow rate of the base stream changed gradually reaching the steady state in a comparable time to the acid one. Overall, the manipulated variables varied without abrupt changes guaranteeing reaching the product quality set-point. Figure 8b shows the trend of command signals going to final control elements of the split-range logic (i.e., gear pumps and electro-actuated valves). When the outlet flow rate reached the split-range value fixed at 1.2 L min^–1^ (dash yellow line), the electro-actuated valve started to close leaving the control action to the gear pump which was able to finely adjust the flow rate in a range of 0.3–1.2 L min^–1^.

After this test, a further experiment was carried out for a set-point variation of the outlet conductivities of −30% concerning the steady-state values (Figure 8c). In this case, the controller reached the set-point value in a time of 32 and 24% higher for the acid and base, respectively. The different behavior between positive and negative set-point variations was attributed to the nonlinearity of the system. Figure 8d depicts the trend of the control action of the electro-actuated valves and the gear pumps for both acid and base. When the split-range condition was reached, the control signals of the pumps were raised to increase the outlet flow rate. Meanwhile, the valves were opened to support the pumps since their action was saturated at 4 V. These results showed how the split-range logic plays a key role in the cascade control to accurately manage the outlet product flow rates in a wide range of 0.3–5.0 L min^–1^.

**Figure 9:**
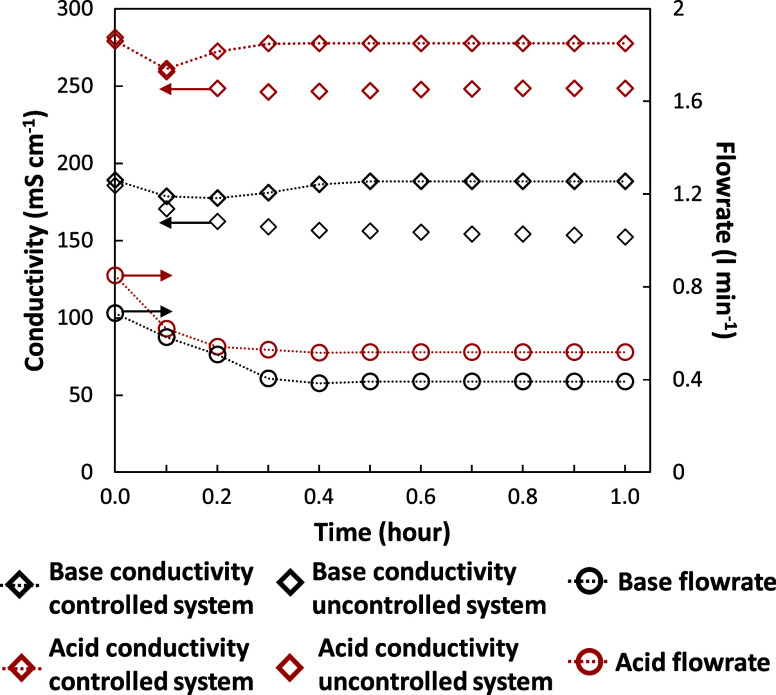
Disturbance response of the composition controller to a sudden variation of −25% in the current density.

To globally evaluate the performance of the composition controller, a disturbance response was tested by suddenly changing the current density provided to the stack. In Figure 9, the trends of the acid and base conductivities are reported for both controlled and uncontrolled systems. It was possible to observe how the steady-state set-point was very well maintained following a very short transitory. Moreover, the prompt response of the controller guaranteed a small deviation of controlled variables (conductivities) from the steady-state condition even during the short transitory, where the peak variation resulted equal to 6 and 5% for the acid and base, respectively, much lower than deviations observed for the uncontrolled system (above 12%). On the other hand, the manipulated variables varied smoothly avoiding any destabilizing effect on the cascade controller.

### 3.2 Operation of the Pilot under Transient PV-Power Supply: Summer Scenario

**Figure 10:**
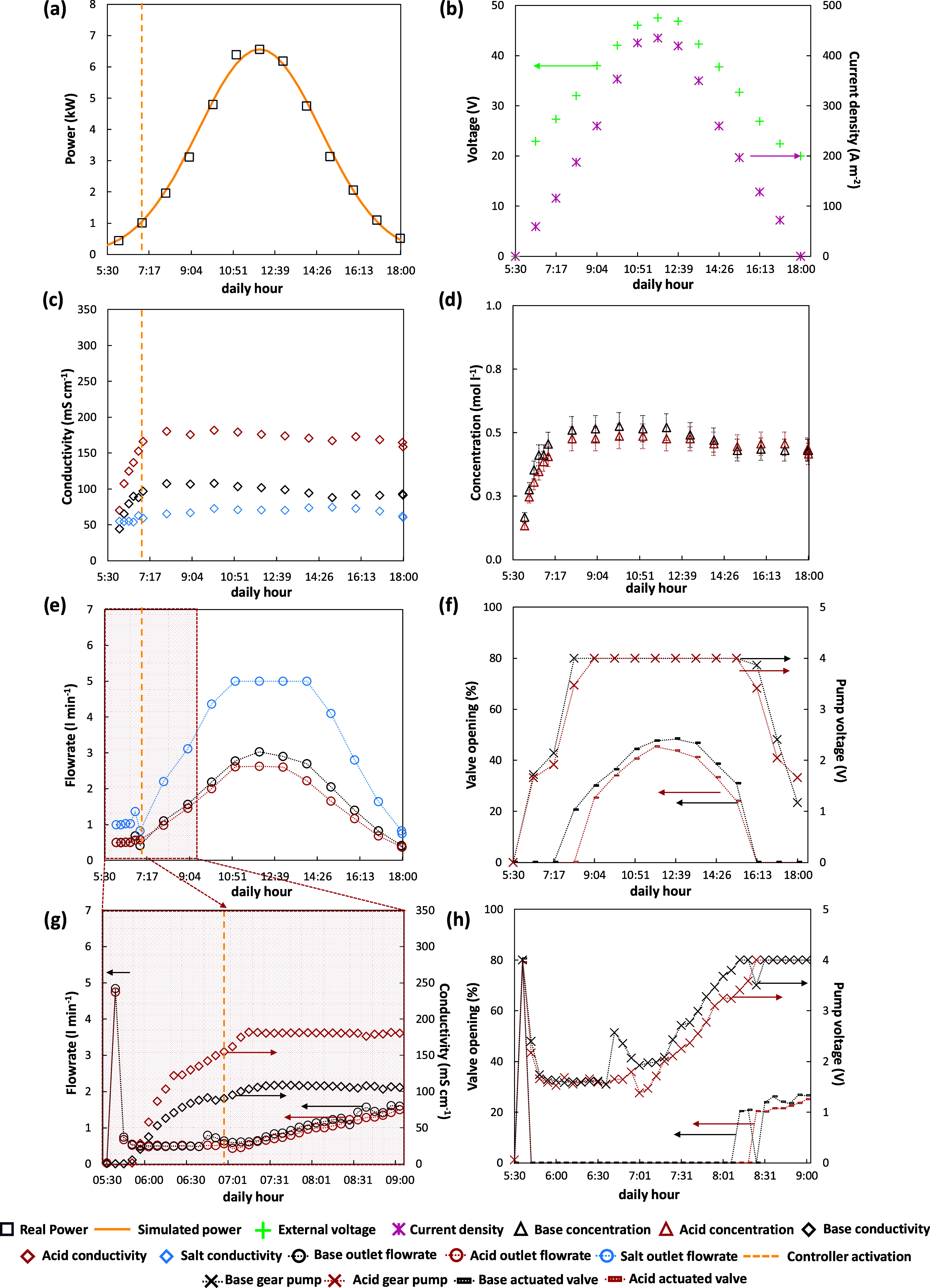
Analysis of main variables trend during the transient operation of the EDBM unit powered by PV in a summer day scenario: (a) real and simulated PV power, (b) applied voltage and current density, (c) outlet conductivities, (d) outlet concentrations, (e) outlet flow rates, (f) electro-actuated valves and gear pumps control signals, (g) zoom of the start-up phase, and (h) valves and gear pumps control signals during the start-up.

To simulate the operation of the EDBM pilot under a transient PV-power supply, a variable power curve was provided to the DC drive controller. Figure 10a reports the power delivered by the power controller in comparison with the real one in a summer scenario on Lampedusa Island (July). It was observed how the power controller was always able to follow the real trend allowing the DC drive to deliver a set-point value corresponding to the maximum power available. However, the daily range of operation was limited to the time slot between 6:00 and 18:00, when the power was 7% of the maximum, to have a minimum current density through the cell pack.

Figure 10b depicts the trends of external voltage and current density. The shape of the curves was qualitatively similar to that of the power set-point. This was linked to the action of the power controller, which varied the manipulated variable (voltage) to delete the offset between the available power and the measured one. Furthermore, no abrupt variations were observed in the external voltage. As a consequence, the EDBM stack was forced to operate in a highly transient regime corresponding to a current density ranging from 50 A m^–2^ at 6:00, in the morning, up to 450 A m^–2^ at noon, when the maximum power peak was reached. The highly dynamic operation of the EDBM during this experimental campaign represents a very unfavorable situation for composition controllers for two reasons:

(a) the control system suffers from continuous disturbance without having time to reach the steady-state conditions;
(b) the wide range of operating current densities could lead to a failure of the control action since the operation of the equipment is highly nonlinear.

Figure 10c shows the trends of the outlet conductivities for the acid, base, and salt solutions, allowing evaluation of the performance of the cascade composition control that worked under these challenging conditions. As can be seen, a satisfactory and reliable response of the control system was obtained: it was found to be capable of keeping the conductivity very close to the steady-state value set at 175 and 100 mS cm^–1^ for acid and base, respectively. These set-point values were set to have a concentration for acid and base solutions of about 0.5 mol L^–1^. This set-point concentration was chosen to analyze relevant industrial scenarios, in which EDBM products can be employed in precipitation (alkaline product)^43,44^ or neutralization processes, as well as in wastewater treatment facilities.^45^Figure 10d reports the concentration trends corresponding to the conductivity trends. The adoption of conductivities as controlled variables allows to keep the product concentrations near the desired targets, notwithstanding the power available changed a lot. The use of outlet conductivity as a controlled variable for managing outlet concentration is a unique feature of this control system. In a previous study conducted by Herrero-Gonzalez et al.,^35^ the pH of the acidic and alkaline streams was used to control the outlet concentrations of EDBM products. However, conventional pH sensors struggle to accurately measure concentrations above 0.1 M for both acids and bases. In contrast, conductivity sensors offer reliable readings because, within the concentration range of this study, an increase in acid or base concentration leads to a nearly proportional increase in conductivity.

The transient behavior occurring in the first part of the graph is related to the control strategy implemented. In particular, the PLC was programmed to hydraulically start the pilot at 5:30. (Figure 10g). In this way, the system was able to reach the hydraulic steady state just before the minimum power from the solar array was available at 6:00. During this initial phase, the cascade control was deactivated, since the power was too low to reach the set-point composition. For this reason, the outlet flow rate of the slave controller was fixed to a value very close to the minimum flow rate that can be delivered by the pump, equal to 0.5 L min^–1^. At 6:00, the available power started to increase as well as the current density supplied to the stack. Thus, at a fixed output flow rate, the conductivity and the concentration of acid and base started to increase until the lower conductivity threshold was reached (dashed yellow line). Then, the master composition controllers were activated setting the outlet flow rates set-point to the minimum value that the gear pumps could provide, equal to 0.4 L min^–1^. After the start-up phase, there was a change in the slope of the conductivity curves, which rapidly reached the steady-state value at 7:00.

The whole trends of the outlet flow rates are reported in Figure 10e for acid, base, and salt streams. The flow rate of the saline solution was always higher in comparison with those of the acid and base ones. This was related to the ratio control between the salt and the base outlet flow rates. In particular, the ratio among these two outlet streams was set to have a double salt flow rate compared to the basic one representing the product with the higher added value. This control strategy allowed us to avoid an excessive impoverishment of the saline solution due to the increase in the acid and base productivities. It was observed that as the available power increased in the first part of the day, the controller raised the outlet flow rates to guarantee the composition set-point for both acid and base, up to a maximum flow rate value once the highest point of the power curve was reached. At the same time, it was appreciated how the flow rates of the products varied gradually without sudden jumps. This phenomenon was attributed to the split-range control implemented in the slave control loop of the cascade controller. Figure 10f shows the behavior of the electrically actuated valve and the gear pump control signals during the whole process. During the initial part of the test, which goes from the hydraulic start-up to 9:00, very low acid and base flow rates were needed, which could not be guaranteed by the control valves as these worked poorly in the condition of almost total closure. This is why, the use of the gear pumps ensured the smooth management of the manipulated variable from 0.4 to 1.2 L min^–1^. Once the required flow rate from the master controller was raised and the split-range condition was exceeded, the gear pump provided its maximum flow rate while the electro-actuated valve started to open to adjust the flow rate in a range between 1.2 and 3 L min^–1^.

**Figure 11:**
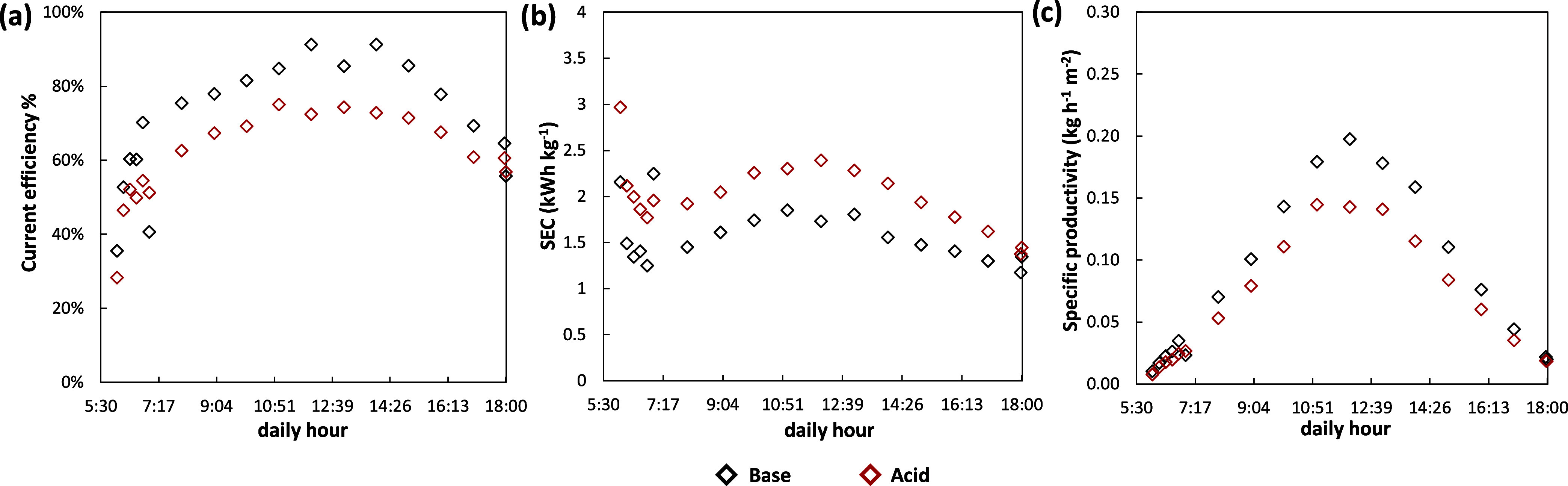
Trends of the main performance indicators during plant operation in a summer day scenario: (a) Current efficiency (CE), (b) Specific energy consumption (SEC), and (c) specific productivity (SP). CE, SEC, and SP are referred to the production of both NaOH and HCl.

The trend of the performance indicators of the process is shown in Figure 11. The current efficiency started from values equal to 30 and 36% for acid and base, respectively, during the initial transient phase where the concentrations were very low. As soon as the set-point concentration was reached, the CE raised, following eq 1, proportionally to the ratio between output flow rate and current density. The maximum current efficiency values were found at the maximum power, equal to 74 and 91% for acid and base, respectively (Figure 11). This difference in CE, between acid and base, is strictly linked to nonideal phenomena such as diffusive fluxes, osmotic fluxes, electro-osmotic fluxes, parasitic currents, and internal leakage affecting the stack performance. These phenomena are emphasized in the acid compartment as part of the produced protons, due to their high mobility, pass through the CEM leading to the acidification of the salt compartments. Nevertheless, high CE values were obtained compared to the existing literature,^46^ underlining how the control system allows for maximization of CE depending on the power availability. The good results obtained in terms of CE were also reflected in the other performance indicators such as SEC and SP. In the first part of the process where CE was very low and the voltage was more or less constant, high values of SEC were obtained, namely, equal to 3.0 and 2.2 kWh kg^–1^ for acid and base, respectively (Figure 11). The minimum SEC values were observed in correspondence with the maximum power, resulting in 1.8 kWh kg^–1^ for the acid and 1.3 kWh kg^–1^ for the base. These results are noticeable if compared to previous SEC values obtained in the literature for EDBM-PV applications. Herrero-Gonzalez et al.^35^ found a best SEC value equal to 4.4 kWh kg^–1^ at 1 M target concentration for the acid product, with a current density of 360 A m^–2^ using FuMa-Tech membranes. While a higher target concentration in EDBM typically leads to higher SEC values, the control strategy employed in this work allowed us to obtain less than halved SEC values for both products with a current density 25% higher with respect to the literature. Additionally, both the CE and SEC values obtained in this work, are comparable to those found in a modeling study^36^ assessing the techno-economic feasibility of coupling EDBM with RES. In terms of SP, the trends were qualitatively similar to CE ones. Once more, the best values of specific productivity were obtained near the maximum power available being 0.14 and 0.2 kg h^–1^ m^–2^ for acid and base, respectively (Figure 11). It was clear that all of the performance parameters for the base were better with respect to the acid. Since the same concentration was obtained for both products, the decrease in performances in the acid was related to the CE which was 13% lower throughout the whole test. This phenomenon was most likely due to the different effects of the acid and base diffusion phenomena in the saline solution channel. As a result of the high mobility of the protons, acid diffusion was presumably dominant.^47^

### 3.3 Operation of the Pilot under Transient PV-Power Supply: Winter Scenario

**Figure 12:**
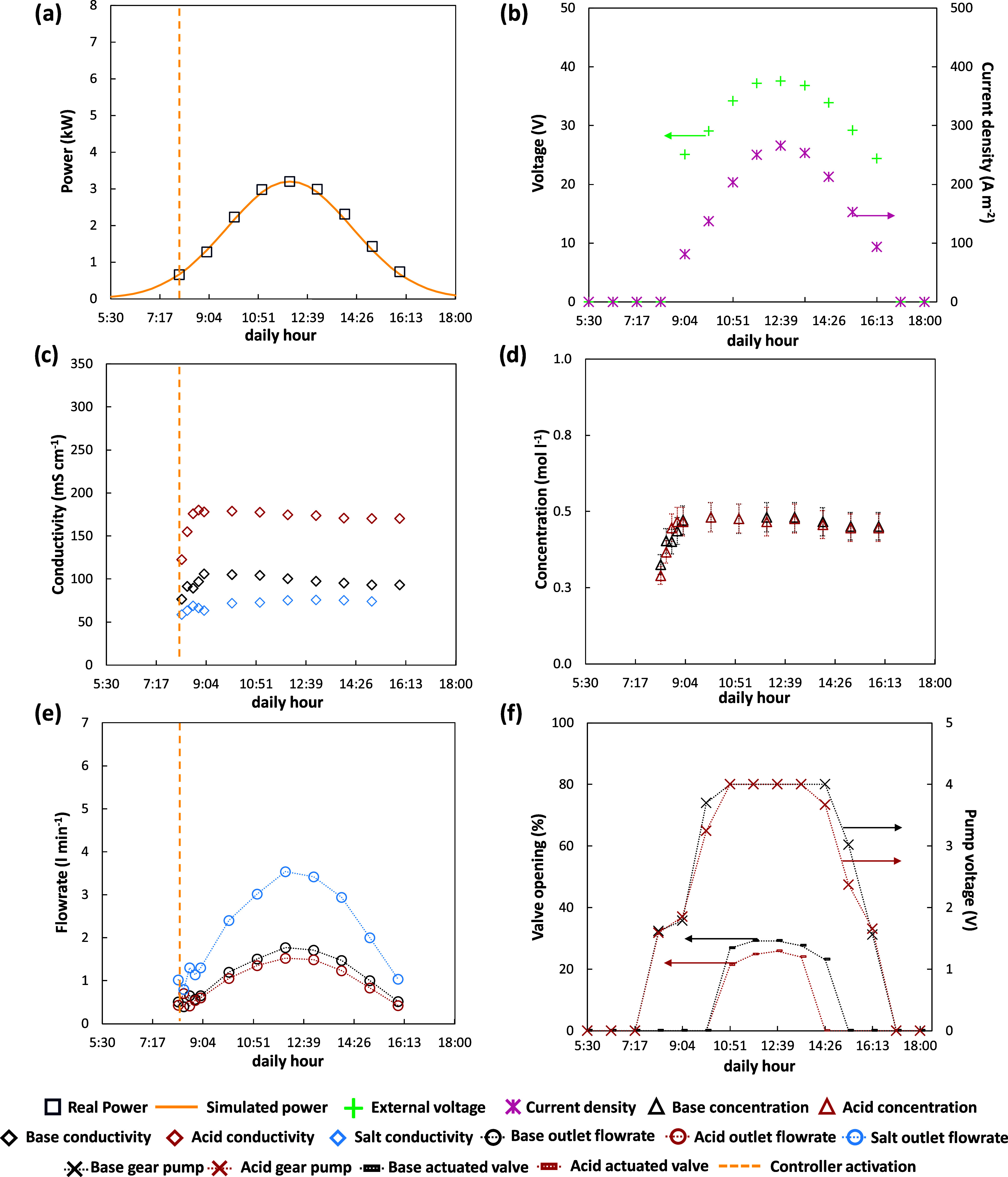
Analysis of main variables trend during the transient operation of the EDBM unit powered by PV in a winter-day scenario: (a) real and simulated PV-power, (b) applied voltage and current density, (c) outlet conductivities, (d) outlet concentrations, (e) outlet flow rates, (f) electro-actuated valves and gear pumps control signals.

The behavior of the EDBM pilot under dynamic regimes was further tested simulating the PV-power availability in a winter scenario in Lampedusa Island (December). Figure 12a reports the trend of the variable DC drive set-point in comparison with the real power available. The operating range was limited to 8 h to have the minimum requested power to start the process, equal to 20% of the maximum one. The behavior of the manipulated variable of the power controller is presented in Figure 12b, showing how the external voltage varied in a tight range to adjust the power. Furthermore, the current density provided to the cell pack ranged from 81 to 265 A m^–2^. As a consequence, the composition controller was able to keep the set-point conductivity values fixed at 175 and 100 mS cm^–1^ for the acid and base, respectively (Figure 12c). The quality of the control action was confirmed by the concentration trends (Figure 12d). Indeed, apart from the start-up phase, a flat trend of concentrations was obtained, equal to 0.5 mol L^–1^ for both acid and base, reflecting the conductivities trend. This demonstrated the effectiveness and reliability of adopting the conductivities of the electrolytic solutions as controlled variables to control the concentration of the products. The trends of the manipulated variable of the cascade controller are reported in Figure 12e. As soon as the power available reached the minimum value (dashed yellow line), the conductivity controller was activated, setting the outlet flow rate at 0.4 L min^–1^ for both acid and base. At 9:00 the target concentrations were reached and therefore the flow rate was raised to keep the controlled variable constant. The manipulated variable showed a maximum in correspondence with the maximum power availability and after 12:00 started to decrease down to 0.42 and 0.52 L min^–1^ for the acid and base, respectively, at 16:00. Also in this scenario, the outlet flow rate of the salt stream was kept twice the base outlet flow rate by the ratio control. Figure 12f shows the control signals of the split-range logic for both gear pumps and electro-actuated valves. As can be seen, the valves were employed solely in the time slot between 11:00 and 14:00 in which the higher value of the outlet flow rates were requested.

**Figure 13:**
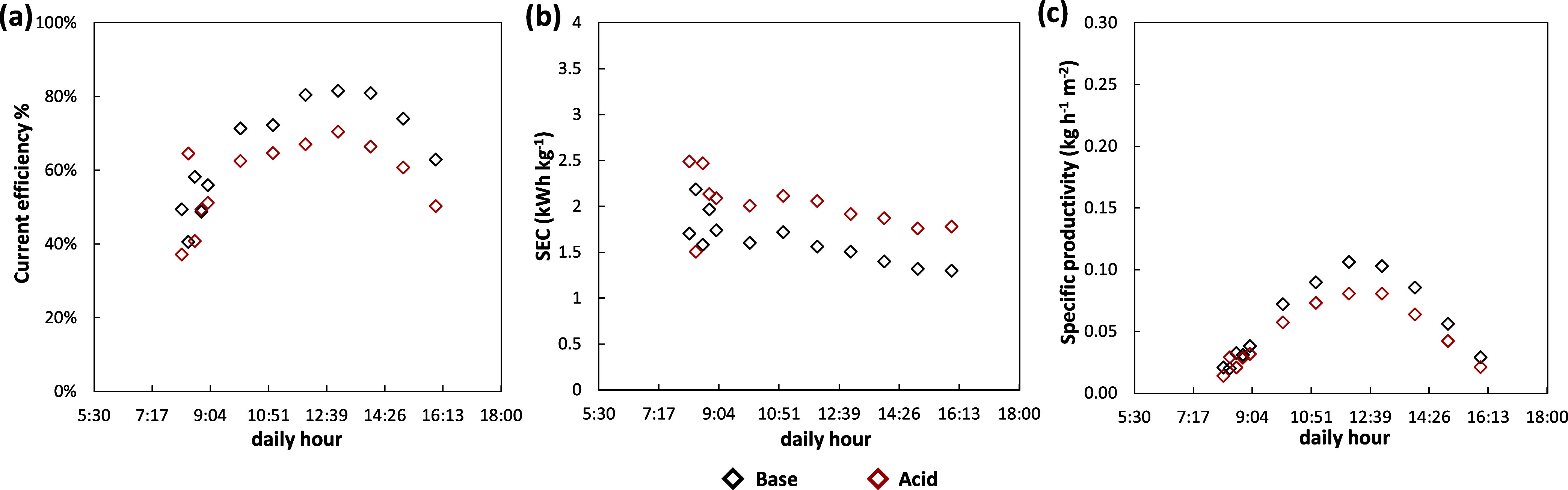
Trends of the main performance indicators during plant operation in a winter-day scenario: (a) Current efficiency (CE), (b) specific energy consumption (SEC), and (c) specific productivity (SP). CE, SEC, and SP are referred to the production of both NaOH and HCl.

The performance indicators of the EDBM pilot operated during the winter scenario are listed in Figure 13. The current efficiency increased slowly during the first part of the test, for both acid and base, due to the small flow rates utilized to bring the concentrations up to the set-point value. When the target was reached, the current efficiency was raised, according to the ratio between the outlet flow rate and the current density, up to 70 and 82%, for acid and base, respectively. On the other hand, the specific energy consumption exhibited a slightly increasing/decreasing trend along the working day, with a stable average value. This behavior was attributed to the tight variation of the external voltage and the outlet flow rates going in the same direction. The lowest values of SEC in this scenario were obtained in the final part of the test in which the power was approximately 23% of the maximum, resulting in 1.8 and 1.3 kWh kg^–1^ of acid and base, respectively. As a consequence of the low product flow rates, small values were found for the SP, ranging between 0.02 and 0.08 kg h^–1^ m^–2^ and between 0.03 and 0.11 kg h^–1^ m^–2^ of acid and base, respectively. In terms of performance indicators, the average results achieved in this work are significantly improved over those obtained with the same EDBM stack in a previous study^48^ with a fixed current density. Specifically, for the base product, an average CE (current efficiency) of 73% was observed, which is considerably higher than the 59.7% CE achieved with a constant current supply. Additionally, the control strategies for managing the available energy led to a lower average SEC (specific energy consumption) of 1.5 kWh kg^–1^ for the alkaline product, compared to the 1.8 kWh kg^–1^ recorded in the previous study.^48^

### 3.4 Comparison between Summer and Winter Scenarios

A comparison between the summer and winter scenarios is reported in this section. The summer scenario, in the month of July, guaranteed the highest peak irradiation in Lampedusa, equal to 980 W m^–2^ at midday. On the other hand, the winter scenario, in the month of December provided the lowest peak irradiation of the year, equal to 600 W m^–2^. Moreover, the sunny hours decreased significantly from 12 h in July down to 8 h in December. Furthermore, a significant drop in the maximum power available was also observed, going from a maximum of 6.5 kWp in July to 3.2 kWp in December. This power reduction played a significant role in the maximum current density that was reached in the winter scenario, equal to 265 A m^–2^.

Despite this, the control system was always able to guarantee the conductivity set-point during the working day for both acid and base. However, in the summer scenario, the power changed rapidly, generating abrupt variations in the current density (disturbance) both before and after the peak value. As a consequence of this, acid and base in the summer scenario reached values slightly higher with respect to the set-point value when the power rose. On the other hand, it was observed a slight reduction in target concentration during the second half of the day (after 12:00), when the power decreased. In fact, Under these conditions, the control system was unable to totally delete the offset since the time required to reach the set-point value was greater than the disturbance variation time. This was confirmed by looking at the central part of the conductivity trends between 11:00 and 13:00. During this time slot, the available power remained almost constant, and consequently, the control system was able to easily cancel the offset. On the other hand, the behavior of the control system in the winter scenario resulted really different. Although the power varied as well, this variation was not sufficient to prevent the controller from maintaining the concentration constantly equal to the set-point value, throughout the working day.

**Figure 14:**
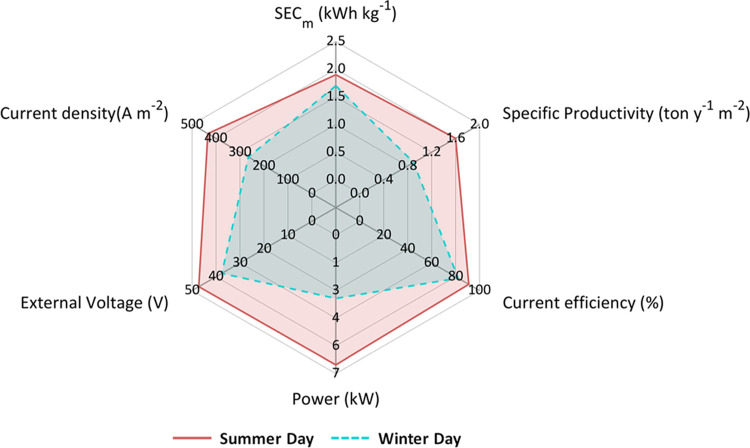
Spider-plot summarizing an overall comparison between the two investigated operating scenarios. All parameters refer to the production of NaOH.

Figure 14 shows an overall comparison of the average parameters obtained in both scenarios, in terms of performance indicators, power, voltage, and current density, for the case of the base, which is the product at a higher added value. The average available power was found to be equal to 3.2 and 2 kW for the summer and winter scenarios, respectively. As a consequence, the DC drive control system supplied an 11% higher external voltage in the summer scenario compared to the winter scenario to seek the available power. Thus, the average value of current density was higher in July (250 A m^–2^) than in December (180 A m^–2^). In terms of CE, considerably high values were obtained in both scenarios (higher than 60%). However, in the summer scenario, CE was 7% higher because the EDBM stack operated in the feed and bleed configuration offered better performance at higher current densities.^48^ Concerning SEC, the same average values were found, likely due to the lower reduction of the average external voltage than the average current efficiency. The last, but not least, important performance parameter was SP. The average SP resulted in 25% lower in the winter scenario with respect to the summer one. This effect was related to the higher values of current density and current efficiency obtained in July, according to eq 3.

These results demonstrated that EDBM technology is perfectly suitable to work in highly transitory regimes such as those pertaining to using renewable energy sources. Moreover, since the plant exhibits better performances with higher energy availability, one might consider a more sophisticated energy supply (i.e., wind energy from smart grids) during the winter season, in which the energy becomes too low, allowing the EDBM system to store more energy as chemical reactants. That way would ensure the stable operation of the system even during periods of low energy availability, guaranteeing continuous and efficient operation by means of enhanced control strategies.

## 4 Conclusions and Future Outlooks

This study has successfully demonstrated the viability of operating an electrodialysis with bipolar membranes (EDBM) plant powered by renewable energy sources under nonstationary conditions, highlighting its efficiency and adaptability for sustainable chemicals production and brine valorization strategies. The implemented control logics such as cascade control, split-range control, ratio control, and override control proved to be effective in maintaining stable product quality and optimizing energy usage, even during transitory regimes. Results under both summer and winter scenarios revealed high current efficiencies (CE), particularly for the base product, with CE values above 80% when operating under favorable conditions. Additionally, the specific energy consumption (SEC) for acid and base production was significantly reduced, reaching values lower than those documented in previous studies, showcasing the potential of EDBM as an energy-efficient technology. Moreover, the behavior of the outlet variables (i.e., concentrations) emphasized that, when enhanced process control strategies are employed, the EDBM can reliably operate in fluctuating energy conditions, while guaranteeing the process target and optimal performance, indicating its suitability for integration into renewable-powered smart grids. However, some performance limitations were observed under lower irradiation conditions, suggesting the need for supplementary energy sources, such as wind power, during low-sunlight periods. In addition, lithium-ion batteries could be integrated as further energy buffers in a higher technology readiness level EDBM plant, to efficiently stabilize the product concentration during the sunrise and sunset phases. This approach could enhance system stability and efficiency, enabling continuous chemical production year-round.

Future research could explore more sophisticated control algorithms to further enhance response precision under highly variable energy supplies (i.e., feedforward control). Additionally, scaling studies are essential to determine the feasibility of industrial-scale applications, particularly in regions where renewable energy conditions are variable. Expanding the system flexibility in handling different waste brine compositions could also maximize the resource recovery potential. With these advancements, EDBM technology will significantly contribute to circular economy goals by valorizing waste streams and supporting sustainable chemical production on a larger scale.

## Supporting Information Available

Description of the pilot unit; schematic representation of the repeating unit of the EDBM technology (triplet) (Figure S1); overview of the main elements composing the EDBM pilot plant (Figure S2); screenshot of the human machine interface developed in LabVIEW environment (Figure S2); list of typical properties of ion-exchange membranes used in EDBM units (Table S1); and list of the instruments and pumps installed in the EDBM pilot plant (Table S2) (PDF)
