# Supplementary material for: Enhanced Schemes for Brine Valorization via Electrodialysis with Bipolar Membranes Powered by Renewable Energy
Source: ACS Omega. 2025 Mar 7;10(10):10122–38. doi: 10.1021/acsomega.4c08609 (PMC11923850; doi:10.1021/acsomega.4c08609)
Supplement: Supplementary file 1 — ao4c08609_si_001.pdf [file ao4c08609_si_001.pdf]

# SUPPORTING INFORMATION

# of the paper titled:

# Enhanced schemes for brine valorisation via ElectroDialysis with Bipolar Membranes powered by Renewable Energy

Calogero Cassaro[1], Giovanni Virruso[1], Andrea Cipollina[1*], Adriano Fagiolini[1], Alessandro Tamburini[1], Giorgio Micale[1]

[1] *Dipartimento di ingegneria, Università degli studi di Palermo, Viale delle scienze Ed. 6, Palermo, 90128, Italy.*

*corresponding author: andrea.cipollina@unipa.it

**Description of the EDBM Pilot**

The EDBM pilot plant consisted of four fundamental parts:

i. The pumping station, which houses the hydraulic circuit as well as the monitoring and control instrumentation;

ii. An electrodialysis stack with bipolar membranes with a total membrane area of 19.2 $m^2$ divided into 40 triplets, supplied by FuMA-Tech GmbH (Germany);

iii. Acquisition and command hardware composed of:

(a) Analog acquisition and command cards, to collect data and provide control signals to actuators;

(b) Chassis where the cards are located;

(c) Computer enabling the operator to interact with the process, through the Human Machine Interface (HMI);

iv. A direct current power supply connected with the EDBM stack.

The pilot scale EDBM plant was designed to work both in discontinuous and continuous mode. However, in this study, only one of these process configurations was tested, namely feed and bleed mode. A more in-depth description of the plant, which is currently the largest investigated in literature so far, has been already presented in a previous work[1].

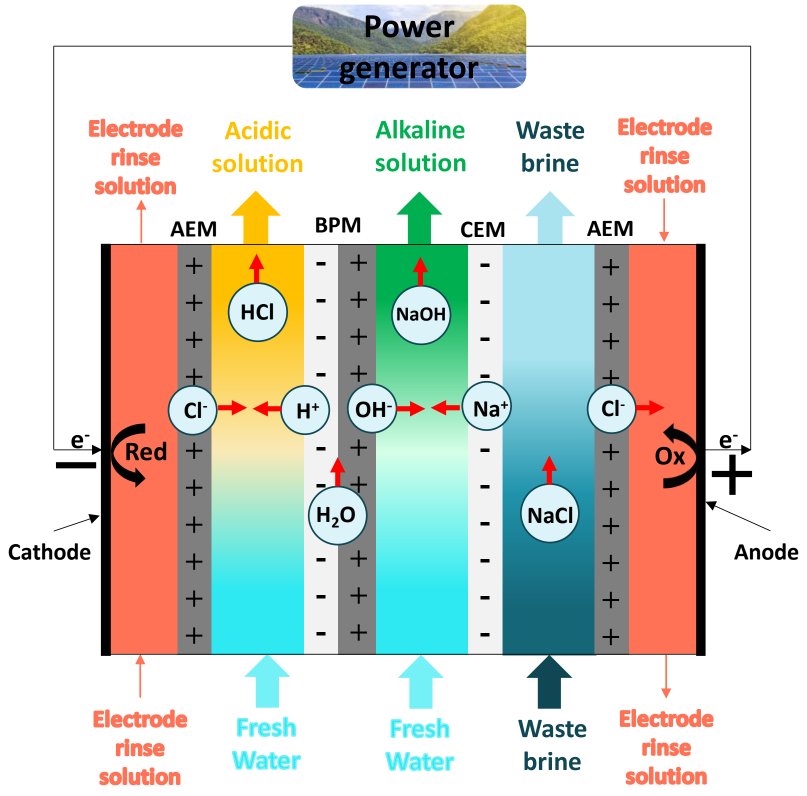


**Figure S1.** Schematic representation of the repeating unit of the EDBM technology (triplet)

**Table S1.** List of typical properties of ion-exchange membranes used in EDBM units.

| Membrane | FAB-PK-130 | FKL-PK-130 | FBM |
|---|---|---|---|
| Reinforcement | PEEK woven web | PEEK woven web | PEEK woven web |
| Resin type | Anion | Cation | Bipolar |
| Thickness | 130 μm | 130 μm | 150 μm |
| Resistance (*) | <8.5 Ohm·cm$^2$ | <10 Ohm·cm$^2$ | n.a. |
| Selectivity (*) | >93% | >98% | n.a. |
| Swelling at 80°C | <2% | <4% | n.a. |
| E-Modulus | >1,000 MPa | >1,000 MPa | n.a. |

(*) Measured in 0.5M NaCl solution

**Table S2.** List of the instruments and pumps installed in the EDBM pilot plant.

| Element | Model | Range | Material | DN (mm) |
|---|---|---|---|---|
| Magnetic induction flowmeter | OPTIFLUX 4100C | 0–30 l min$^{-1}$ | PTFE | 20 |
| Conductivity meter | OPTISENS IND 1000 | 1–2000 mS cm$^{-1}$ | PP | 20 |
| pH meter | SMARTPAT PH 8320 | 0–14 | Glass AH | 15 |
| Pressure transducer | OPTIBAR P 1010 C | 0–6 bar | AISI 316L | 15 |
| Turbine pump | TEOREMA PTM 2.5x6 | 0–3,500 rpm | PP | 25 |
| Gear Pump | TEOREMA FG-300 | 0–1,6 l min$^{-1}$ | AISI 316L | 8 |
| Electrically Actuated Valve | FIP VKDIVEL020E0E | 0-100% | PP | 20 |

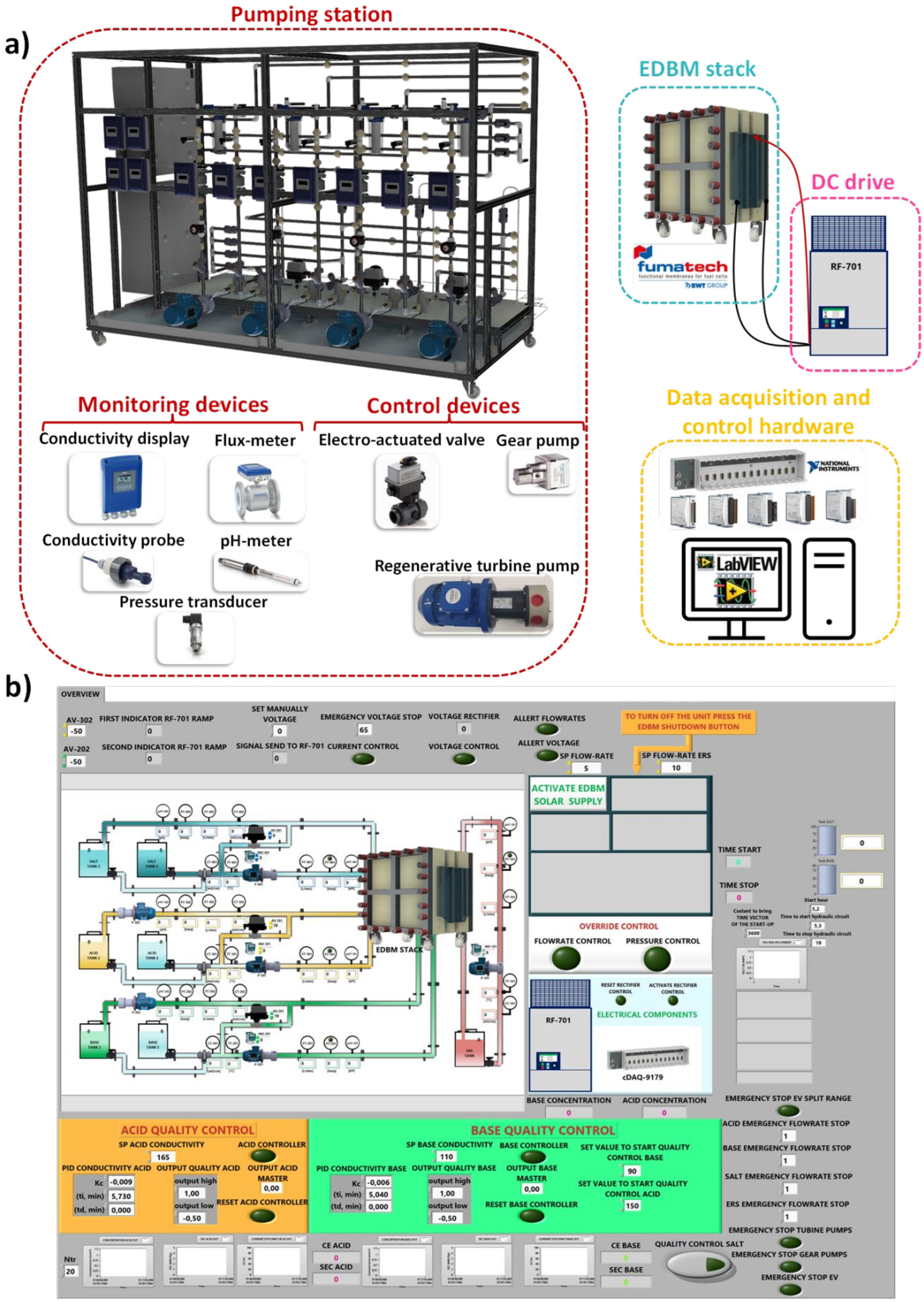


**Figure S2.** a) Overview of the main elements composing the EDBM pilot plant; b) Screenshot of the Human Machine interface developed in LabVIEW environment.

*S1 Acquisition and command hardware and software*

All input and output signals to/from the process were managed via National Instruments (NI) hardware and software tools. The data acquisition hardware consisted of a chassis (NI cDAQ-9179), acquisition cards (C series: NI-9203, NI-9208) and command cards (C series: NI-9264, NI-9265 and NI-9266). LabVIEW software (National Instrument) was used for the development of the programmable logic controller (PLC) and of the Human Machine Interface (HMI).

## References

(1) Cassaro, C.; Virruso, G.; Culcasi, A.; Cipollina, A.; Tamburini, A.; Micale, G. Electrodialysis with Bipolar Membranes for the Sustainable Production of Chemicals from Seawater Brines at Pilot Plant Scale. *ACS Sustain. Chem. Eng.* **2023**, *11* (7). https://doi.org/10.1021/acssuschemeng.2c06636.
